# Editorial Comment: A 16-yr Follow-up of the European Randomized study of Screening for Prostate Cancer

**DOI:** 10.1590/S1677-5538.IBJU.2020.03.06

**Published:** 2020-02-20

**Authors:** J Hugosson, MJ Roobol, M Månsson, TLJ Tammela, M Zappa, V Nelen, Felipe Lott

**Affiliations:** 1 University of Göteborg Institute of Clinical Sciences Department of Urology Göteborg Sweden Department of Urology, Institute of Clinical Sciences, Sahlgrenska Academy at the University of Göteborg, Göteborg, Sweden; 2 Erasmus Medical Centre Rotterdam Netherlands Erasmus Medical Centre, Rotterdam, The Netherlands; 3 University of Göteborg Institute of Clinical Sciences Department of Urology Göteborg Sweden Department of Urology, Institute of Clinical Sciences, Sahlgrenska Academy at the University of Göteborg, Göteborg, Sweden; 4 University of Tampere Faculty of Medicine and Life Sciences Tampere Finland University of Tampere, Faculty of Medicine and Life Sciences, Tampere, Finland; 5 ISPRO Oncological network, Prevention, and Research Institute Florence Italy ISPRO, Oncological network, Prevention, and Research Institute, Florence, Italy; 6 Provinciaal Instituut voor Hygiëne Antwerp Belgium Provinciaal Instituut voor Hygiëne, Antwerp, Belgium; 1 Instituto Nacional do Câncer – INCA Rio de JaneiroRJ Brasil Instituto Nacional do Câncer – INCA, Rio de Janeiro, RJ, Brasil

## COMMENT

This is the 16 years follow-up of the European Randomized study of Screening for Prostate Cancer (ERSPC) that was initiated in 1993 and previously published with 9, 11, and 13 years of follow-up (1–3). This trail try to elucidate the effect of regular prostate-specific antigen (PSA) screening on prostate cancer (PCa) mortality.

This paper shows that the absolute reduction in PCa mortality still increases with longer follow-up, while the relative risk reduction remains at 20% since the initial report (1–3). There is still a 41% excess incidence in the screening arm. The median follow-up from diagnosis is modest (8.8 years in the screening arm and 5.4 years in the control arm) given the natural course of PCa.

The number needed to diagnose for averting one PCa death was 18 in this update paper and was much higher in the previous ones.

This high level evidence publication shows that the absolute effect of screening on PCa mortality increases with longer follow-up.

## References

[1] 1. Schröder FH, Hugosson J, Roobol MJ, Tammela TL, Ciatto S, Nelen V, et al. Screening and prostate-cancer mortality in a randomized European study. N Engl J Med. 2009;360:1320-8. Schröder FH, Hugosson J, Roobol MJ, Tammela TL, Ciatto S, Nelen V, et al. Prostate-cancer mortality at 11 years of follow-up. N Engl J Med. 2012;366:981-90. 10.1056/NEJMoa0810084 19297566

[2] 2. Schröder FH, Hugosson J, Roobol MJ, Tammela TL, Zappa M, Nelen V, et al. Screening and prostate cancer mortality: results of the European Randomised Study of Screening for Prostate Cancer (ERSPC) at 13 years of follow-up. Lancet. 2014;384:2027-35. 10.1016/S0140-6736(14)60525-0 PMC4427906 25108889

